# Recent advances in targeting the cGAS–STING pathway for immunotherapy in orthopedic diseases

**DOI:** 10.3389/fimmu.2025.1726423

**Published:** 2025-12-03

**Authors:** Feipeng Wu, Shan Hui, Dapeng Li, Guoqing Pan, Wenchao Zhang

**Affiliations:** 1 Department of Spine Surgery, Affiliated Hospital of Jiangsu University, Zhenjiang, Jiangsu, China; 2 Department of Oncology, Zhenjiang First People’s Hospital, Zhenjiang, Jiangsu, China; 3 Institute for Advanced Materials, School of Materials Science and Engineering, Jiangsu University, Zhenjiang, Jiangsu, China; 4 Department of Orthopedics, First People’s Hospital of Jintan, Changzhou, China

**Keywords:** cGAS-STING pathway, orthopedic diseases, innate immunity, bone remodeling, immunotherapy

## Abstract

With increasing evidence highlighting the role of immune dysregulation in orthopedic diseases such as osteoarthritis, osteoporosis, intervertebral disc degeneration, delayed fracture healing, and bone tumors, the cyclic GMP-AMP synthase–stimulator of interferon genes (cGAS–STING) pathway has emerged as a central mediator of innate immune sensing and sterile inflammation. This review summarizes the core mechanisms of cGAS-STING activation and its involvement in regulating inflammatory responses, bone remodeling, cell death, and immune cell polarization in musculoskeletal tissues. In particular, we discuss recent findings on how aberrant cGAS-STING signaling contributes not only to chronic inflammatory bone loss but also to the tumor immune microenvironment in primary bone cancers and bone metastases. Furthermore, we highlight the therapeutic prospects of targeting this pathway using agonists or inhibitors, which show promise for novel immunomodulatory approaches in the treatment of orthopedic diseases, including skeletal malignancies.

## 1 Introduction

With population aging and lifestyle changes, the incidence of orthopedic disorders—including osteoarthritis(OA), rheumatoid arthritis(RA), osteoporosis(OP), intervertebral disc degeneration(IVDD), and impaired fracture healing—continues to rise, severely compromising patients’ quality of life and imposing a substantial socioeconomic burden. Accumulating evidence indicates that chronic inflammation and immune dysregulation are key drivers of the onset and progression of these conditions; processes such as articular cartilage degradation, bone loss, and delayed tissue repair are tightly linked to immune pathways (1).

The cyclic GMP–AMP synthase (cGAS)–stimulator of interferon genes (STING) pathway is a central innate immune sensor of cytosolic DNA. Upon sensing DNA, cGAS catalyzes the synthesis of cyclic GMP–AMP(cGAMP), which activates STING and subsequently engages downstream TANK-binding kinase 1 (TBK1)–interferon regulatory factor 3(IRF3) and NF-κB signaling, inducing the production of type I interferons(IFN-I) and pro-inflammatory cytokines (2, 3). Initially regarded as primarily mediating antiviral innate immunity, the cGAS–STING axis has more recently been recognized as a critical contributor to sterile inflammation, cellular senescence, autoimmunity, and tissue injury (4, 5).

In orthopedic diseases, aberrant activation of the cGAS–STING pathway has been linked to articular chondrocyte apoptosis, osteoclast activation, senescence of intervertebral disc cells, and delayed fracture repair (6, 7). For example, in IVDD models, the release of mitochondrial DNA(mtDNA) into the cytosol can activate cGAS–STING signaling, provoking pro-inflammatory responses and accelerating apoptosis (6); In OP, pathway activation is closely associated with inflammatory dysregulation of the bone marrow microenvironment and may exacerbate bone loss by altering macrophage polarization and disrupting the osteoblast/osteoclast balance (7).

These insights suggest that therapeutic modulation of the cGAS–STING pathway may offer new immunotherapeutic avenues for orthopedic disease. Proposed strategies include using STING inhibitors to mitigate local joint inflammation and employing STING agonists to enhance antitumor immune responses in bone malignancies (8, 9).

Accordingly, this review will summarize the fundamental mechanisms of the cGAS–STING pathway and systematically examine its roles in representative orthopedic conditions (OA, RA, OP, IVDD, and OS), as well as discuss potential therapeutic strategies, with the aim of informing immune-based interventions for orthopedic disorders.

## 2 The basic mechanism of the cGAS–STING pathway

### 2.1 Discovery of the cGAS–STING signaling pathway

The discovery of the cGAS–STING pathway represents a significant milestone in the study of innate immunity, revealing the core mechanism by which cells sense cytosolic DNA and initiate immune responses. In 2008, researchers first identified STING as an endoplasmic reticulum–localized transmembrane protein capable of inducing type I interferon production even in the absence of exogenous receptor activation, indicating its pivotal role in noncanonical antiviral defense (10).

However, STING itself does not directly recognize DNA, and its upstream activation mechanism remained elusive at that time. In 2013, cGAS was identified as the first enzyme capable of directly sensing cytosolic double-stranded DNA(dsDNA). Upon binding to DNA, cGAS catalyzes the synthesis of the cyclic dinucleotide second messenger cGAMP, which subsequently binds to and activates STING. This activation triggers the TBK1–IRF3 signaling cascade, leading to the expression of IFN-I and proinflammatory cytokines (11). This discovery not only established cGAS as a key DNA sensor in innate immunity but also unveiled cGAMP as a natural secondary messenger in immune signaling.

Subsequent studies revealed that endogenous mammalian cGAMP contains mixed 2′–5′ and 3′–5′ phosphodiester linkages (referred to as 2′3′-cGAMP), which confers higher binding affinity and biological activity toward STING (12). The elucidation of this mechanism filled a major gap in our understanding of intracellular DNA recognition and provided new theoretical insights into antiviral defense, tumor immunity, and autoimmune inflammation.

### 2.2 Structural characteristics of cGAS and STING

#### 2.2.1 Structure of cGAS

cGAS is a pivotal immune sensor enzyme, approximately 60 kDa in size, that recognizes exogenous cytosolic dsDNA and catalyzes the synthesis of the cyclic dinucleotide cGAMP from ATP and GTP, thereby activating the STING signaling pathway and initiating a cascade of immune responses (13). Structurally, cGAS comprises an N-terminal domain and a C-terminal catalytic domain, the latter containing a nucleotidyltransferase (NTase) core and a Mab21 domain that together adopt a bilobed configuration centered around the catalytic core (5, 14). Although originally thought to reside primarily in the cytoplasm, cGAS has also been detected in the nuclear compartment, suggesting its broader regulatory roles (15).

The cGAS protein contains multiple structural domains, each playing an essential role in its function. The N-terminal extension domain, which is rich in positively charged residues, interacts electrostatically with the negatively charged phosphate backbone of DNA. This region is non-conserved and approximately 130–150 amino acids in length, providing flexibility that enables cGAS to accommodate and recognize DNA molecules of diverse lengths and conformations. The Mab21 domain constitutes the highly conserved catalytic core responsible for the generation of cGAMP, a critical step in initiating immune signaling. The NTase domain facilitates the catalytic reaction and mediates DNA binding, thereby activating cGAS enzymatic activity. In the absence of DNA, the bilobed catalytic domain remains in an inactive conformation; upon DNA binding, it undergoes conformational rearrangement that promotes cGAMP synthesis. The coordinated action of these structural domains allows cGAS to dynamically sense and respond to cytosolic DNA, effectively triggering downstream immune responses (16, 17).

Dimerization of cGAS represents a key activation step in dsDNA recognition. In its basal state, cGAS exists as a monomer, but upon binding to dsDNA, conformational changes promote dimer formation, which exposes the catalytic site and initiates cGAMP production. This process results in the assembly of a 2:2 cGAS–DNA complex, wherein two cGAS molecules interact with two segments of dsDNA to form a stable, active complex. Dimerization and subsequent 2:2 complex formation enhance the specificity and efficiency of DNA recognition, ensuring robust activation of the innate immune response. Through this mechanism, cGAS enables cells to rapidly and accurately detect cytosolic DNA and mount an effective defense against external threats (18, 19).

#### 2.2.2 Structure of STING

STING is a dimeric transmembrane adaptor protein located on the endoplasmic reticulum(ER) membrane, responsible for sensing cytosolic cyclic dinucleotides (CDNs), such as cGAMP, and initiating the production of IFN-I (20, 21). The human STING protein consists of 379 amino acid residues and is organized into three main regions: an N-terminal transmembrane domain (TMD; residues 1–138) comprising four transmembrane helices, a cytoplasmic ligand-binding domain (LBD; residues 139–336), and a C-terminal tail (CTT; residues 337–379) (22, 23).

The TMD, located at the N-terminus, anchors STING to the ER membrane through its four transmembrane helices and facilitates STING dimerization, which represents the initial step in its activation. The LBD, positioned on the cytoplasmic side, adopts a butterfly-shaped dimeric structure, forming a deep V-shaped cleft between two subunits that specifically binds cyclic dinucleotides such as cGAMP. Ligand binding induces conformational rearrangements in STING, promoting oligomerization and subsequent activation of downstream signaling. The CTT at the C-terminus stabilizes higher-order STING oligomers and mediates interactions with TBK1 kinase and the IRF3 transcription factor, ultimately leading to the transcriptional induction of IFN-I. The coordinated action of these three structural domains ensures the critical role of STING in immune signaling, particularly in antiviral defense and antitumor immunity (21, 24–26).

Higher-order STING oligomerization is essential for its functional activation. STING dimers associate through hydrophobic interactions and disulfide bonds to form multimeric assemblies, which facilitate the recruitment and activation of TBK1. Activated TBK1 subsequently phosphorylates IRF3, driving the transcription of IFN-I and other immune response genes (27, 28). This hierarchical activation mechanism underscores the structural precision and regulatory complexity of the STING pathway in innate immune signaling.

### 2.3 The cGAS–STING pathway

Under normal physiological conditions, DNA is strictly confined within the nucleus of eukaryotic cells, while extranuclear DNA is rapidly degraded by nucleases such as three-prime repair exonuclease 1 (TREX1) and ribonuclease H2 (RNase H2). When abnormal accumulation of cytosolic dsDNA occurs, cGAS recognizes and binds to these DNA molecules, leading to its activation (29–31). Such aberrant cytosolic DNA may originate from viral or bacterial infections, or from endogenous sources such as necrotic cells, mitochondrial damage, and genomic instability (32–34).

Upon binding to these dsDNA molecules, cGAS undergoes a conformational change that enables it to catalyze the conversion of adenosine triphosphate (ATP) and guanosine-5′-triphosphate (GTP) into cGAMP, a potent immunostimulatory second messenger that directly activates STING (35). Upon cGAMP binding, STING undergoes structural rearrangement and translocates as a dimer from the ER to the perinuclear Golgi apparatus, initiating a cascade of downstream signaling events. During this process, STING aggregates around the nucleus and facilitates the activation of TBK1. Activated TBK1 subsequently phosphorylates IRF3, which translocates to the nucleus to induce the transcription of type I interferon(IFN-I) genes (25, 36–38). In parallel, STING also activates the NF-κB signaling pathway through interaction with IκB kinase (IKK) and NF-κB–inducing kinase (NIK), promoting the release of various proinflammatory cytokines, including TNF-α, IL-6, and IFN-I. The NF-κB and TBK1–IRF3 pathways act synergistically to enhance the expression of IFN-I. Subsequently, IFN-I binds to its heterodimeric receptor IFNAR1/IFNAR2, activating Janus kinase 1 (JAK1), which phosphorylates members of the signal transducer and activator of transcription (STAT) family. This activation induces the transcription of interferon-stimulated genes (ISGs), forming a positive feedback loop that amplifies IFN-I production and strengthens the host’s immune defense (38–41). IFN-I exert broad immunostimulatory functions, promoting the maturation, migration, and activation of various immune cells such as dendritic cells (DCs), T cells, and natural killer (NK) cells (42, 43).

**Figure 1:**
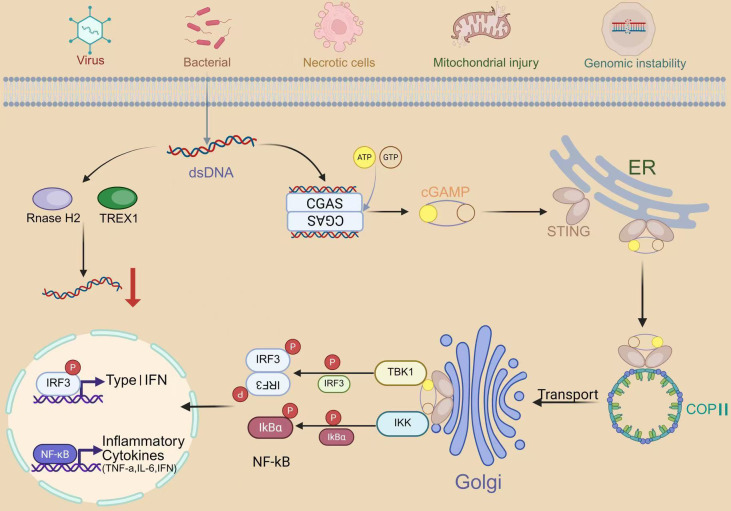
The cGAS–STING pathway and regulation of cytosolic DNA sensing. Cytosolic double-stranded DNA (dsDNA) generated during infection by DNA viruses or bacteria, or released after sterile damage (necrotic cells, mitochondrial injury) and genomic instability, is detected by cGAS. Binding of dsDNA activates cGAS to catalyze the formation of 2′3′-cyclic GMP–AMP (2′3′-cGAMP) from ATP and GTP. cGAMP engages ER-resident STING, which then exits the ER in COPII-coated vesicles and traffics to the Golgi apparatus. Activated STING recruits TBK1 and the IKK complex, leading to phosphorylation of IRF3 and IκBα, respectively. Phosphorylated IRF3 dimerizes and translocates into the nucleus to induce type I interferon (IFN) genes, while IκBα phosphorylation and degradation liberate NF-κB (p65/p50) to drive transcription of inflammatory cytokines such as TNF-α and IL-6 (and additional IFN-stimulated genes). RNase H2 and TREX1 degrade cytosolic DNA, thereby limiting pathway activation. Black arrows denote activation/trafficking; the red arrow indicates degradation; “P” marks phosphorylation events. (Illustration created with Biorender).

Beyond its immune functions, the cGAS–STING pathway is closely associated with DNA damage repair and cellular senescence (44). Recent studies have shown that persistent activation of the cGAS–STING pathway may accelerate cellular senescence, whereas its inhibition under certain conditions can enhance DNA damage repair, thereby helping maintain normal cellular homeostasis (45) (**Figure 1**).

## 3 The role of the cGAS–STING pathway in orthopedic diseases

### 3.1 The cGAS–STING pathway in OA

Osteoarthritis(OA) is a chronic musculoskeletal disorder in which biomechanical stress plays a pivotal role, predominantly affecting elderly individuals (46). The disease is characterized by progressive joint pain, stiffness, and loss of mobility, severely impairing patients’ quality of life. OA is a multifactorial and complex disease influenced by aging, environmental factors, and biological mechanisms (47–50). Immune cells—particularly activated neutrophils, macrophages, and T lymphocytes—play crucial roles in driving inflammation within the affected joints. Proinflammatory cytokines such as IL-1β, TNF-α, and IL-6, along with chemokines, further promote immune cell recruitment, amplifying the inflammatory cascade (51–53).

During OA pathogenesis, factors such as mechanical stress, inflammatory cytokines (e.g., IL-1β), and cellular senescence lead to cell damage, apoptosis, or senescence, resulting in the release of nuclear or mtDNA into the cytoplasm (54). For instance, abnormal mechanical loading can activate the mechanosensitive ion channel Piezo1, inducing mitochondrial Ca^2+^ overload and subsequent mtDNA leakage (55). Moreover, recent evidence indicates that ER stress and the unfolded protein response (UPR) can also enhance cytosolic DNA accumulation (56). These leaked DNA fragments act as damage-associated molecular patterns (DAMPs), which are recognized by cGAS, thereby triggering activation of the STING pathway (57).

Upon recognition of cytosolic dsDNA, cGAS catalyzes the synthesis of cyclic cGAMP, which functions as a second messenger that binds and activates STING. Activated STING induces downstream signaling cascades involving IRF3 and NF-κB, leading to the production of IFN-I and proinflammatory cytokines, thus initiating an innate immune response. Studies have shown that STING expression is upregulated in chondrocytes from OA patients. Excessive activation of STING promotes the expression of matrix metalloproteinases (MMP13) and ADAMTS5, key catabolic enzymes responsible for the degradation of cartilage matrix components such as type II collagen and aggrecan (58). Moreover, STING activation suppresses the synthesis of anabolic cartilage matrix proteins (e.g., aggrecan and type II collagen) while promoting cellular senescence and apoptosis in chondrocytes (59).

Mechanistically, STING mediates chondrocyte apoptosis and senescence through activation of the NF-κB pathway, thereby exacerbating cartilage degeneration. Conversely, inhibition of STING-dependent NF-κB signaling attenuates chondrocyte inflammation, synovitis, cellular senescence, and matrix degradation (60). In temporomandibular joint osteoarthritis (TMJOA) models, mechanical stress–induced mtDNA leakage activates the cGAS–STING pathway, promoting glycolysis in chondrocytes, suppressing matrix anabolism, and enhancing catabolic activity, collectively accelerating cartilage destruction (61).

**Figure 2:**
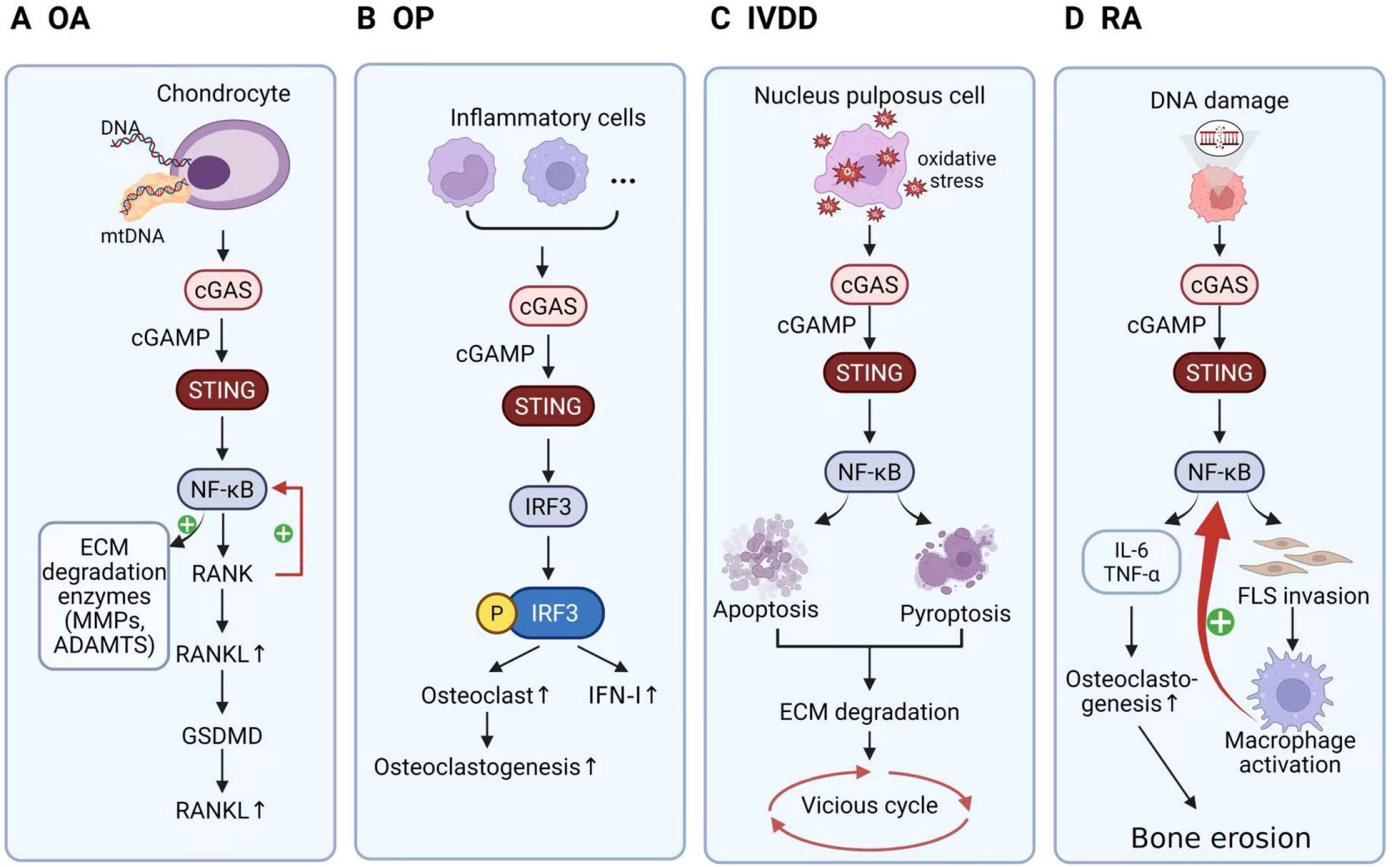
Roles of the cGAS–STING pathway in musculoskeletal and joint disorders. In all panels, cytosolic DNA sensed by cGAS generates cGAMP to activate STING and its downstream effectors. **(A)** Osteoarthritis (OA). In chondrocytes, mtDNA/DNA activates cGAS–STING, leading to NF-κB signaling. NF-κB induces extracellular matrix–degrading enzymes (MMPs, ADAMTS) and up-regulates the RANK/RANKL axis; GSDMD-linked pyroptotic signaling is implicated, together contributing to cartilage matrix breakdown and increased RANKL. **(B)** Osteoporosis (OP). In inflammatory cells, cGAS–STING signaling promotes IRF3 phosphorylation and type I interferon (IFN-I) production, favoring osteoclast increase and enhanced osteoclastogenesis. **(C)** Intervertebral disc degeneration (IVDD). Oxidative stress in nucleus pulposus cells activates cGAS–STING→NF-κB, driving apoptosis and pyroptosis, which accelerates extracellular matrix degradation and sustains a vicious cycle of disc degeneration. **(D)** Rheumatoid arthritis (RA). DNA damage triggers cGAS–STING→NF-κB signaling, inducing IL-6 and TNF-α, promoting FLS invasion and macrophage activation, increasing osteoclastogenesis, and culminating in bone erosion.Arrows indicate activation/flow; “P” denotes phosphorylation. OA, osteoarthritis; OP, osteoporosis; IVDD, intervertebral disc degeneration; RA, rheumatoid arthritis; ECM, extracellular matrix; MMPs, matrix metalloproteinases; ADAMTS, a disintegrin and metalloproteinase with thrombospondin motifs; FLS, fibroblast-like synoviocytes; IFN-I, type I interferon; NF-κB, nuclear factor κB; IRF3, interferon regulatory factor 3; GSDMD, gasdermin D; RANK, receptor activator of NF-κB; RANKL, receptor activator of NF-κB ligand.

Beyond cartilage metabolism, STING activation also contributes to OA-associated pain, partly by modulating peripheral sensitization molecules. Pharmacological inhibition of STING effectively alleviates mechanical allodynia associated with OA (62, 63). Additionally, targeting proteasome activity to enhance STING ubiquitination and degradation has been shown to ameliorate both age-related and trauma-induced OA in experimental models (1, 64) (**Figure 2****)**.

### 3.2 The cGAS–STING pathway in RA

Rheumatoid arthritis(RA) is a prototypical systemic autoimmune disorder characterized by persistent synovial inflammation, aberrant proliferation and invasion of fibroblast-like synoviocytes (FLS), and progressive joint destruction (63, 65, 66). Increasing evidence indicates that cGAS–STING signaling pathway plays a pivotal role in the pathogenesis of RA, bridging innate immune sensing with chronic inflammation and autoimmunity (67, 68).

In the synovial tissue and FLSs of RA, abnormal cytosolic accumulation of dsDNA and mtDNA has been observed. This accumulation is attributed to mitochondrial dysfunction, dysregulated mitochondrial fission, release of nuclear or mitochondrial nucleic acids during apoptosis and necrosis, and defective autophagy or mitophagy, as well as microenvironmental stressors such as hypoxia and nutrient deprivation (69–72). Such nucleic-acid accumulation has been demonstrated to activate the cGAS/STING signaling pathway, which subsequently triggers the TBK1–IRF3 and NF-κB cascades, leading to enhanced production of IFN-I and multiple proinflammatory cytokines, including IL-6, TNF-α, and IL-1β (68, 73). These mechanisms collectively establish a persistent cycle of “defective nucleic acid clearance–aberrant immune sensing–inflammatory amplification, “ which forms the molecular basis for sustained activation of the cGAS–STING signaling pathway in RA.

Evidence from patient-derived cells and animal models indicates that activation of the cGAS–STING pathway promotes mitochondrial reactive oxygen species (ROS) generation and modulates the MST1–FOXO1 signaling axis, thereby enhancing the migration and invasive potential of FLSs and contributing to cartilage destruction (73, 74);In addition, macrophage extracellular traps (METs) and neutrophil extracellular traps (NETs) released into the synovial microenvironment contain self-DNA that can act as persistent ligands for cGAS activation. DNA released from these extracellular traps triggers PI3K/Akt signaling in FLSs, leading to tumor-like proliferation and invasiveness of synovial fibroblasts (75).

Clinical and translational studies further reveal that circulating cell-free DNA (cfDNA) levels are elevated in RA patients, accompanied by increased expression of cGAS and STING in both synovial and peripheral immune cells. Moreover, proinflammatory cytokines such as TNF-α exacerbate mtDNA accumulation by suppressing cytosolic DNase II activity and impairing mitophagy, thereby sustaining a self-reinforcing loop of nucleic-acid signaling and chronic inflammation (68).

From a therapeutic perspective, experimental studies demonstrate that pharmacological inhibition of the cGAS–STING pathway or enzymatic degradation of cytosolic DNA significantly reduces proinflammatory cytokine production, attenuates the invasive behavior of FLSs, and alleviates arthritis severity in preclinical models (76, 77).

Collectively, these findings establish aberrant activation of the cGAS–STING pathway as a crucial mechanistic bridge linking innate immune sensing of self-DNA to pathological reprogramming of synovial cells. This pathway acts in concert with classical immune mechanisms—such as T/B-cell activation, cytokine cascades, and the JAK/STAT and NF-κB pathways—and represents a promising therapeutic target for the treatment of RA (68, 73, 74) (**Figure 2**).

### 3.3 The cGAS–STING pathway in OP

Osteoporosis (OP) is a chronic metabolic bone disorder characterized by decreased bone mass, microarchitectural deterioration, and increased bone fragility, predisposing individuals—especially postmenopausal women and the elderly—to fractures in sites such as the spine, hip, and radius (78). Beyond its metabolic nature, OP is increasingly recognized as an inflammatory condition, in which systemic and local inflammation disrupts bone remodeling homeostasis (79, 80). The hallmark pathological features include thinning and loss of trabeculae, enlarged marrow cavities, and decline in bone mineral density (BMD), all resulting from an imbalance between bone formation and bone resorption (81).

Emerging evidence suggests that the cGAS–STING signaling pathway plays a pivotal role in regulating bone resorption and immune-mediated bone remodeling. In OP, chronic low-grade inflammation leads to cellular senescence, apoptosis, and the release of cytosolic DNA, which in turn activates cGAS. Upon activation, cGAS catalyzes the production of cyclic cGAMP, which binds and activates STING, triggering downstream NF-κB signaling (82). This cascade promotes the expression of receptor activator of NF-κB ligand (RANKL), a key osteoclastogenic cytokine that stimulates osteoclast differentiation and activation, thereby accelerating bone loss (83). In addition to its direct role in osteoclastogenesis, the cGAS–STING pathway influences immune cell function in the bone microenvironment. In ovariectomized (OVX) mouse models, increased formation of neutrophil extracellular traps (NETs)—a process termed NETosis—has been observed. These NETs can activate macrophage-derived RAW264.7 cells via the cGAS–STING–AKT2 pathway, promoting osteoclast differentiation and exacerbating bone resorption (84). Moreover, STING activation has been shown to inhibit the formation of type H vessels, a specialized capillary subtype critical for coupling angiogenesis and osteogenesis, suggesting that STING may also impair bone formation by suppressing angiogenic support (85). Interestingly, the effects of STING activation appear context-dependent. In certain conditions, STING-induced interferon-β (IFN-β) signaling suppresses c-Fos expression and inhibits NFATc1 activation, thereby restraining osteoclastogenesis and reducing bone resorption (84). These findings indicate that the cGAS–STING pathway may exert dual and sometimes opposing effects on bone metabolism—promoting bone loss under chronic inflammatory states while offering protection through IFN-β–mediated regulation in other contexts.

From a therapeutic perspective, pharmacological inhibition of the cGAS–STING pathway has been shown to effectively attenuate bone resorption and ameliorate bone loss, suggesting its potential as a promising therapeutic target for OP (86). However, it is important to note that STING signaling exhibits pleiotropic downstream effects: while NF-κB activation promotes inflammation and osteoclastogenesis, the IFN-β branch may counteract bone loss by inhibiting osteoclast formation. Thus, global STING inhibition may act as a double-edged sword—reducing inflammation and bone loss on one hand, but potentially compromising its regulatory role in bone homeostasis on the other (63). Future research should aim to selectively modulate distinct STING signaling branches to maximize therapeutic efficacy while minimizing adverse effects (**Figure 2**).

### 3.4 The cGAS–STING pathway in IVDD

Intervertebral disc degeneration (IVDD) is one of the leading causes of chronic low back pain and disability, particularly prevalent among elderly populations (87). The pathological hallmarks of IVDD include senescence of nucleus pulposus cells (NPCs), degradation of the extracellular matrix (ECM), cell apoptosis, chronic inflammation, and structural deterioration of the intervertebral disc. These degenerative changes ultimately result in the loss of disc function, compromising spinal stability and mobility (88).

In IVDD, mechanical stress, oxidative stress, and genomic instability can induce damage to nuclear or mtDNA, leading to their leakage into the cytoplasm. These cytosolic dsDNA fragments(cytoDNA) are recognized and bound by cGAS, triggering activation of the cGAS–STING signaling pathway (89). Upon activation, the pathway stimulates IRF3 and NF-κB signaling, promoting the senescence-associated secretory phenotype (SASP) and thereby accelerating the degenerative process of the intervertebral disc (89, 90).

The cGAS–STING pathway also contributes to inflammatory cell death through activation of the NLRP3 inflammasome, inducing both pyroptosis and apoptosis. For instance, overexpression of STING enhances IRF3 activation, leading to increased apoptosis of NPCs and aggravation of disc degeneration (91, 92). In addition, aberrant accumulation of R-loops—three-stranded nucleic acid structures—has been shown to induce dsDNA release, activate the cGAS–STING pathway, and promote NPC senescence and IVDD. Restoring normal R-loop homeostasis significantly attenuates cGAS–STING activation and delays cellular senescence (88).

Therapeutically, small-molecule inhibitors such as H-151, LDK378, and CMA have been shown to effectively suppress STING activity, reducing NPC senescence and apoptosis and slowing IVDD progression (90). Similarly, siRNA-mediated knockdown of STING markedly decreases inflammatory cytokine levels, alleviates cellular senescence and apoptosis, and delays disc degeneration (92). Moreover, studies have revealed that autophagy plays a key role in regulating STING homeostasis: in healthy NPCs, STING is degraded via autophagy to maintain low basal expression and suppress inflammation. However, in senescent NPCs, impaired autophagic degradation leads to STING accumulation, thereby promoting inflammation and cellular aging. Thus, restoring STING autophagy-mediated degradation may represent a novel therapeutic approach for IVDD (90).

In summary, IVDD is a multifactorial degenerative disorder involving complex cellular and molecular mechanisms. The cGAS–STING pathway plays a crucial role in IVDD progression by promoting cellular senescence, inflammatory responses, and cell death. Pharmacological or genetic inhibition of this pathway effectively mitigates disc degeneration, highlighting it as a promising therapeutic target (63). Nevertheless, further studies are required to explore specific and safe therapeutic interventions targeting this pathway and to evaluate their clinical applicability in IVDD management (**Figure 2**).

### 3.5 The cGAS–STING pathway in OS

Osteosarcoma (OS) is one of the most common primary malignant bone tumors, characterized by marked genomic instability, high invasiveness, and a strong propensity for pulmonary metastasis (93). The rapid accumulation of DNA damage and chromosomal aberrations leads to the presence of abundant cytosolic DNA, which provides the foundation for activation of the cGAS–STING signaling pathway (45). As a cytosolic DNA sensor, cGAS recognizes these aberrant DNA fragments and catalyzes the synthesis of the cyclic dinucleotide 2′3′-cGAMP, which subsequently activates STING. This activation triggers phosphorylation of TBK1 and IRF3, inducing the expression of type I interferons (IFN-I) and chemokines such as CCL5 and CXCL10. These mediators promote dendritic cell (DC) maturation, enhance antigen presentation, and drive CD8^+^ T-cell infiltration, thereby converting the immunologically “cold” OS microenvironment into an immune-active “hot” tumor, which enhances the antitumor immune response (94–96).

However, the expression and functionality of STING vary significantly among different OS cell lines. For instance, U2OS and SAOS-2 cells show limited responsiveness to exogenous cGAMP stimulation, whereas MG63 and LM6 cells retain partial responsiveness (97). Radiation therapy not only directly induces DNA damage but also enhances cytosolic DNA accumulation, further activating the cGAS–STING pathway. In STING-deficient cells, radiation-induced upregulation of chemokines is markedly attenuated, suggesting that this pathway serves as a crucial molecular bridge between radiotherapy and antitumor immune activation (98).

Notably, the cGAS–STING pathway exerts a “double-edged sword” effect in OS. Chronic or aberrant activation can lead to sustained inflammation, upregulation of PD-L1, and infiltration of myeloid-derived suppressor cells (MDSCs), all of which contribute to immune tolerance and tumor immune evasion (95, 99). Moreover, tumor-derived exosomes and the surface enzyme ENPP1 can hydrolyze extracellular cGAMP, thereby disrupting paracrine STING signaling and generating adenosine, which suppresses antitumor immunity and promotes tumor metastasis (100, 101). Thus, STING activation alone may not be sufficient for durable antitumor responses, and combinatorial strategies involving immune checkpoint inhibitors, ENPP1 inhibitors, or metabolic modulators may be required to achieve sustained therapeutic benefit. Preclinical studies have demonstrated that intratumoral administration of STING agonists in OS models elicits strong local and systemic immune responses and synergizes with PD-1/CTLA-4 blockade (20). In addition, SGLT2 inhibitors have been shown to activate the STING/IRF3/IFN-β axis in murine OS models, suppressing tumor growth and synergizing with 2′3′-cGAMP to enhance antitumor efficacy (102). These findings suggest that combining STING agonists with metabolic agents, radiotherapy, or chemotherapy may significantly improve the efficacy of OS immunotherapy.

**Figure 3:**
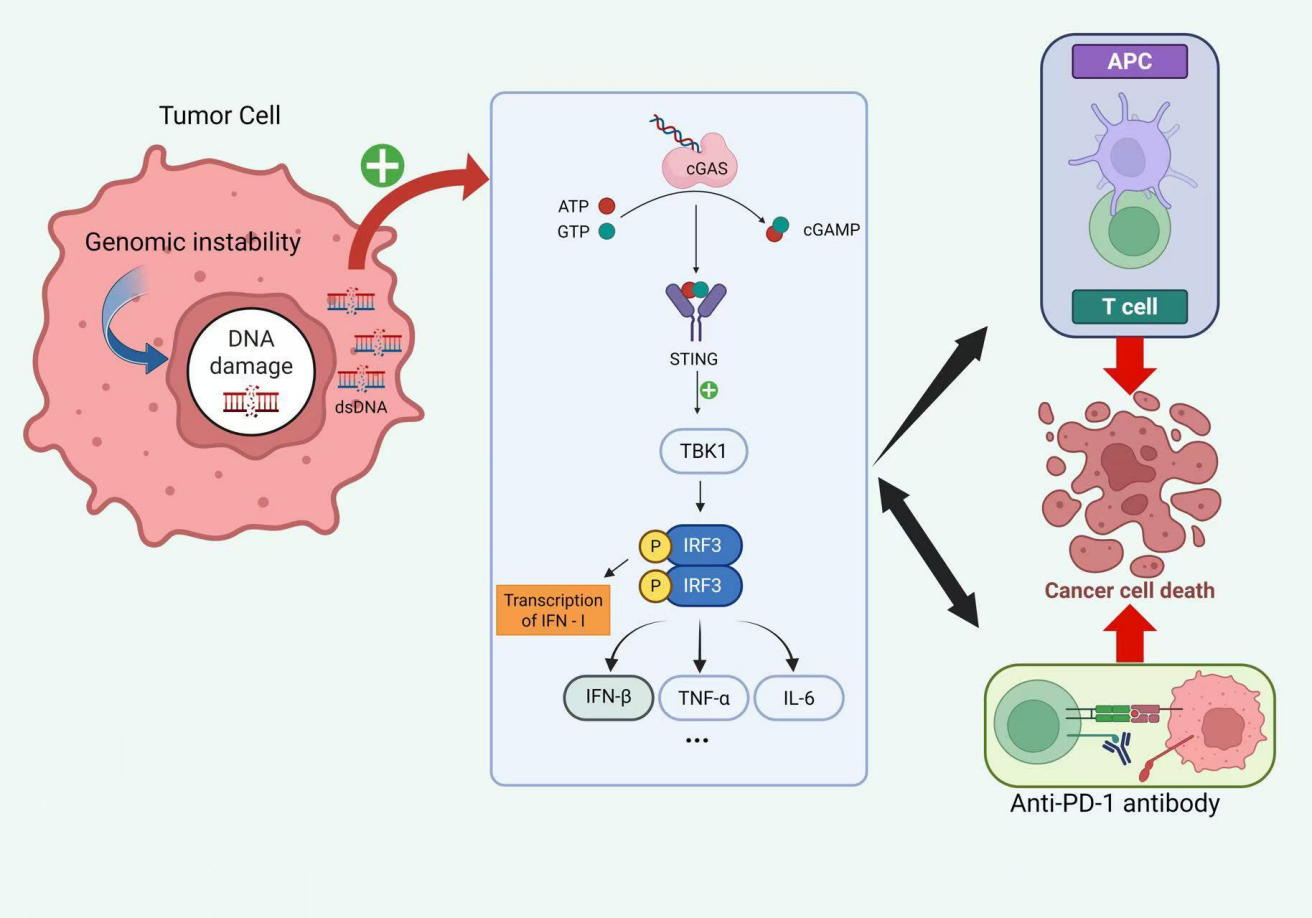
cGAS–STING signaling links tumor genomic instability to antitumor immunity and response to PD-1 blockade. Genomic instability in tumor cells generates cytosolic double-stranded DNA (dsDNA), which activates cGAS to synthesize 2′3′-cGAMP from ATP and GTP. cGAMP engages ER-resident STING, leading to TBK1 recruitment and IRF3 phosphorylation and dimerization. Activated IRF3 drives transcription of type I interferons (e.g., IFN-β) and interferon-stimulated genes, while cGAS–STING signaling can also promote NF-κB–dependent cytokines such as TNF-α and IL-6. The resulting interferon and inflammatory milieu enhances antigen presentation by APCs and primes effector T cells, culminating in cancer cell death. Therapeutic PD-1 blockade further augments T-cell–mediated cytotoxicity, acting synergistically with cGAS–STING–driven antitumor immunity. “P” denotes phosphorylation; “+” indicates activation; arrows indicate signaling/functional flow. cGAS, cyclic GMP–AMP synthase; cGAMP, cyclic GMP–AMP; STING, stimulator of interferon genes; ER, endoplasmic reticulum; TBK1, TANK-binding kinase 1; IRF3, interferon regulatory factor 3; APC, antigen-presenting cell; IFN, interferon; NF-κB, nuclear factor κB; PD-1, programmed cell death protein 1.

In summary, the cGAS–STING signaling pathway plays a dual role in OS, functioning as both an essential antitumor immune mechanism and a potential mediator of immune escape. A deeper understanding of the multilayered regulatory functions of this pathway within OS cells and the tumor microenvironment will provide a solid theoretical foundation for developing more precise and effective immunotherapeutic strategies against OS (**Figure 3**).

### 3.6 Immunotherapeutic implications of the cGAS–STING pathway in orthopedic disease

Although prior sections primarily focused on signaling and pathophysiology, this subsection emphasizes the immunotherapeutic implications of cGAS–STING activation across orthopedic contexts.

The cGAS–STING axis is not only a key instigator of innate immune sensing and activation but also provides a clear mechanistic foundation for immunotherapy across disease contexts. In oncology, both nucleotide and non-nucleotide STING agonists enhance dendritic-cell priming, induce type I interferon responses, and convert “cold” tumors into immune-inflamed microenvironments, thereby synergizing with immune checkpoint inhibitors and improving efficacy (42, 103–105). These advances can be extrapolated to orthopedic disorders characterized by sterile inflammation and aberrant tissue remodeling. In OA, cGAS–STING–mediated inflammatory amplification and remodeling of the synovial–cartilage immune milieu support context-dependent modulation within an immunotherapeutic framework; in parallel, extracellular vesicle (EV)–based delivery enables joint-localized administration of cGAS–STING modulators (106), and recent RA-focused immunomodulation work further substantiates the therapeutic rationale (107). In OP, STING-driven type I interferon and inflammatory cues influence the osteoclast–osteoblast balance; accordingly, targeting STING may recalibrate bone remodeling via immune reprogramming and reduce fragility risk (108). In OS, activation of cGAS–STING can enhance antigen presentation and recruit T cells into the bone tumor microenvironment, providing a mechanistic basis for combinations with chemotherapy/radiotherapy or checkpoint blockade (109). Given that sustained or excessive STING activation may elicit paradoxical immunosuppression, T-cell exhaustion, tissue toxicity, and imbalance of skeletal remodeling, translational application should implement disease- and stage-specific pathway control—selecting agonism or inhibition as appropriate—together with dose–exposure control and delivery optimization to maximize the benefit–risk profile (42, 105). At the practical level, controlled-release systems, targeted carriers, and local delivery can optimize exposure–response relationships, limit off-target inflammation, and strengthen tissue specificity (104).

## 4 Drug development targeting the cGAS–STING pathway

The cGAS–STING signaling pathway plays a pivotal role in the onset and progression of various orthopedic diseases. In disorders such as OP and arthritis, overactivation of the pathway often triggers chronic inflammation and accelerates disease progression. For these inflammation-driven conditions, inhibiting the enzymatic activity of cGAS or STING may represent an effective therapeutic strategy to reduce excessive immune activation and alleviate clinical symptoms.

Conversely, STING agonists have shown promising progress in the orthopedic field, particularly in enhancing bone fracture healing and antitumor immune responses. Activation of the STING pathway can strengthen local immune responses and promote bone repair and remodeling. Although specific cGAS agonists remain in the early stages of investigation, their therapeutic potential cannot be overlooked. A growing number of preclinical studies targeting cGAS or STING selectively have reported encouraging results, laying a solid foundation for future clinical translation and offering novel strategies for innovative orthopedic therapies.

### 4.1 cGAS inhibitors

cGAS inhibitors can be broadly divided into four major categories. The first category consists of competitive inhibitors targeting the catalytic pocket, such as RU.521, which directly binds to the catalytic site of cGAS and competes with substrates like ATP and GTP. This prevents the synthesis of 2′, 3′-cGAMP and subsequently downregulates the downstream STING–TBK1–IRF3 signaling cascade and type I interferon expression (110).

The second category includes molecules that disrupt cGAS–DNA binding, such as Suramin, which displaces bound DNA and disrupts the cGAS–DNA complex, thereby blocking activation (111); Another example, the cyclic peptide XQ2B, specifically targets the DNA-binding surface of cGAS, preventing its liquid–liquid phase separation (LLPS) on DNA and thus inhibiting the formation of active signaling condensates (112). These inhibitors act at an earlier stage of the signaling process by interfering with the DNA sensing step.

The third category involves nucleic acid-based inhibitors, represented by A151 (TTAGGG oligonucleotide), which mimics DNA and competitively binds to cGAS without activating its enzymatic function. This competitive interaction blocks the generation of endogenous cGAMP and subsequent interferon release (113).

The fourth category includes chemically modifying inhibitors, such as aspirin, which acetylates key lysine residues of cGAS (Lys384, Lys394, Lys414). This acetylation alters cGAS conformation, preventing DNA binding and maintaining the protein in an inactive state, thereby suppressing self-DNA–induced inflammation and autoimmunity under TREX1-deficient conditions (114).

In summary, catalytic-pocket inhibitors (e.g., RU.521) act directly at the enzymatic core to block product synthesis, DNA-binding inhibitors (e.g., Suramin, XQ2B, A151) interrupt the DNA recognition process, and chemically modifying drugs (e.g., aspirin) indirectly modulate enzyme activity by altering protein structure. These diverse mechanisms represent multi-dimensional approaches for pharmacological modulation of the cGAS–STING pathway, providing a foundation bfor the development of selective and clinically viable small-molecule inhibitors (**Table 1**).

**Table 1:** The cGAS inhibitors.

Agent	First posted data	Conditions	Mechanism	Main observed effects	Status	NCT number
RU.521	2017	Autoimmune diseases	Competitive inhibitor of the cGAS catalytic site(mouse selective), blocking ATP/GTP binding and cGAMP synthesis	Decreased Type I IFN and IL-6 in macrophages; reduced joint inflammation in mouse autoimmune models	Preclinical	N/A
PF-06928215	2017	Cancer	Inhibits cGAS by binding to its catalytic site, thereby blocking substrate DNA binding and suppressing the enzymatic synthesis of cGAMP.	Decreased IRF3 phosphorylation; reduced tumor-associated inflammation *in vitro*	Preclinical	N/A
G140/G150	2019	Autoimmune diseases	Human-selective inhibitors targeting the cGAS enzymatic pocket	Suppressed IFN-β response and inflammation in SLE models	Preclinical	N/A
TDI-6570	2021	Neurodegenerative diseases	Inhibits cGAS–DNA complex formation and cGAMP synthesis	Ameliorated neuroinflammation and neuronal loss in Alzheimer’s mouse model	Preclinical	N/A
XQ2B	2023	Autoimmune diseases	Covalent cGAS inhibitor targeting catalytic cysteine residues	Decreased Cytokine expression *in vitro*; alleviated systemic inflammation in mice	Preclinical	N/A
Suramin	2019	Cancer, viral infections	Non-specific DNA-binding blocker that prevents cGAS activation	Inhibits cGAMP formation;Decreased IFN-β in virus-infected cells	Preclinical	N/A
A151	2004	Cancer	Synthetic oligodeoxynucleotide that competes with dsDNA for cGAS binding	Decreased IFN-β, TNF-α, IL-1β; attenuated immune activation in tumor and autoimmune models	Preclinical	N/A
Aspirin	2019	Cancer prevention, pain	Aspirin directly acetylates cGAS and reduces cGAMP synthesis	Decreased type I IFN signaling; improved disease phenotypes in AGS mice	Marketed	N/A (repurposed drug)

### 4.2 STING inhibitors

STING inhibitors can be classified into three main types. The first type includes covalent inhibitors, such as C-176 and H-151, which form irreversible covalent bonds with key cysteine residues on STING (e.g., Cys91), blocking its palmitoylation and oligomerization. This inhibits STING activation and suppresses the downstream TBK1–IRF3 signaling cascade and type I interferon production (86, 115). C-176 is a reversible covalent inhibitor that targets STING Cys91, while H-151, an optimized derivative, binds irreversibly to achieve more stable inhibition of STING activation (116).

The second type comprises competitive inhibitors of the ligand-binding pocket, which occupy the cyclic dinucleotide (CDN) binding site of STING and compete with endogenous agonists such as 2′, 3′-cGAMP or exogenous CDNs, thereby preventing pathway activation (117). A representative compound, SN-011, exhibits higher binding affinity than natural ligands and effectively suppresses STING activation, significantly reducing cytokine release in various inflammation models (118).

The third type includes STING degraders, designed to reduce protein abundance rather than merely inhibit activity. These compounds typically promote proteasome- or lysosome-dependent degradation of STING, leading to sustained inhibition of downstream signaling (86). A representative example is P8, a nitrofuran-derived PROTAC-like compound that possesses both inhibitory and degradative properties. In THP-1 cells, P8 mediates lysosome-dependent STING degradation with a 24-hour DC_50_ of approximately 2.58 μM, markedly suppressing STING-dependent interferon responses. *In vivo*, P8 exhibits renoprotective effects in cisplatin-induced acute kidney injury models, highlighting the therapeutic potential of degrader-based strategies (119).

Overall, covalent inhibitors (e.g., C-176, H-151) block STING activation by modifying critical residues, competitive inhibitors (e.g., SN-011) interfere at the ligand recognition stage, and degraders (e.g., P8) achieve durable suppression by reducing protein abundance. These mechanisms illustrate the multidimensional strategies available for targeting STING, laying a foundation for the development of selective and clinically promising inhibitors. Many of these molecules have demonstrated significant efficacy in animal models of acute kidney injury, pulmonary inflammation, psoriasis, and neuroinflammation, underscoring their therapeutic potential for inflammatory and autoimmune diseases (**Table 2**).

**Table 2:** The STING inhibitors.

Agent	First posted data	Conditions	Mechanism	Main observed effects	Status	NCT number
C-176	2018	Autoimmune diseases, inflammatory diseases	Covalent modifier of STING Cys91;mouse-selective; blocks activation-induced palmitoylation and oligomerization	Decreased type I interferon signaling and inflammatory cytokines in mouse models.	Preclinical	N/A
H-151	2018	Autoimmune diseases, inflammatory diseases	Covalent STING antagonist at Cys91; blocks palmitoylation; active on human and mouse STING	Reduced TBK1/IRF3 activation and type I interferon responses in human cells and *in vivo*.	Preclinical	N/A
C-178	2018	Inflammatory diseases	Covalent binding to Cys91; mouse-selective; inhibits palmitoylation and TBK1 recruitment	Decreased IFN-β reporter activity; inhibited STING-dependent signaling in mouse systems.	Preclinical	N/A
SN-011	2021	Autoimmune diseases, inflammatory diseases	Non-covalent antagonist that binds the CDN pocket and locks STING in an inactive/open conformation	Lowered type I interferon and pro-inflammatory cytokines; blocked activation by 2’3’-cGAMP/HSV/SAVI mutants.	Preclinical	N/A
DWL-4-140	2023	Inflammatory diseases	Interacts with STING CDN-binding domain (CBD); inhibits STING multimerization and signaling (mouse-biased)	Decreased DNA-induced IFN-β responses and inflammatory factors *in vitro* and *in vivo*.	Preclinical	N/A
Astin C	20198	Cancer, inflammatory diseases	Natural cyclopeptide that binds STING CTD and suppresses cGAS-STING activation (affects STING–TBK1 axis)	Reduced type I interferon signaling; anti-inflammatory and anti-tumor effects in models.	Preclinical	N/A
P8 (STING-IN-10)	2024	Inflammatory diseases	PROTAC-like dual agent: in THP-1 cells induces lysosome-dependent STING degradation; in RAW264.7 cells acts primarily as an inhibitor	Attenuated STING-driven inflammatory readouts; cell-type-dependent degrader versus inhibitor behavior.	Preclinical	N/A

### 4.3 STING agonists

STING agonists can generally be divided into three major categories. The first category comprises cyclic dinucleotide (CDN) analogues, such as ADU-S100 (also known as MIW815) and MK-1454, which mimic endogenous CDNs like 2′, 3′-cGAMP. These compounds directly bind STING, induce its oligomerization and conformational rearrangement, and promote its translocation from the ER to the Golgi and other intracellular membranes, thereby activating the TBK1–IRF3 signaling cascade and inducing type I interferon and proinflammatory cytokine expression (120). These agents have strong activation potency and mimic natural mechanisms, but their high hydrophilicity, poor *in vivo* stability, and limited tissue permeability restrict their use mainly to intratumoral administration (121).

The second category consists of non-nucleotide small-molecule agonists, which mimic the binding interactions of natural CDNs but without a nucleotide backbone, allowing improved pharmacokinetic properties and systemic delivery. For example, M335, identified through high-throughput screening and surface plasmon resonance (SPR) assays, effectively activates the TBK1–IRF3–IFN pathway *in vitro (*122). Another representative compound, SR-717, activates multiple STING alleles across species and induces type I interferon responses and antitumor effects *in vivo (*123). These molecules can be administered intravenously or orally and upregulate IFN-β, TNF-α, and other cytokines *in vivo*, inhibiting tumor growth, promoting immune cell infiltration, and establishing antitumor immune memory.

The third category encompasses delivery-enhanced agonist systems, including exoSTING and lipid-encapsulated CDN formulations. Encapsulation of STING agonists in exosomes or cationic liposomes enhances their accumulation and stability in antigen-presenting cells while reducing systemic toxicity (124). For instance, DOTAP/cholesterol liposome–encapsulated ADU-S100 demonstrates improved serum stability and enhanced STING activation in antigen-presenting cells compared with free drug formulations (121).

In conclusion, STING agonists—whether CDN analogues, non-nucleotide small molecules, or delivery-enhanced systems—activate the STING–TBK1–IRF3/NF-κB pathway to induce type I interferons and inflammatory cytokines, enhance antigen presentation, and promote CD8^+^ T-cell–mediated immunity. Preclinical and early clinical studies have demonstrated not only their tumor-suppressive potential but also synergistic effects when combined with immune checkpoint inhibitors such as PD-1/PD-L1 antibodies (120). Furthermore, STING agonists have shown enhanced efficacy when combined with CTLA-4 inhibitors, chemotherapy or radiotherapy (e.g., carboplatin plus anti–PD-1 in ovarian cancer models (125)), PARP inhibitors, innate immune adjuvants (e.g., TLR9 agonists (126)), and oncolytic viruses. Nanodelivery platforms have further expanded their utility in overcoming PD-1–resistant tumors (127). Thus, combinatorial therapeutic strategies involving STING agonists hold promise for improving the immunotherapeutic response rate, overcoming resistance, and optimizing clinical outcomes in OS and other malignancies (**Table 3**).

**Table 3:** The STING agonists.

Agent	First posted data	Conditions	Mechanism	Main observed effects	Status	NCT number
ADU-S100 (MIW815)	2016	Cancer	CDN analog that binds/activates human STING	Increased type I interferon and cytokines in tumor microenvironment; immune-cell infiltration; early clinical activity in IT studies.	Clinical	NCT02675439
MK-1454	2018	Cancer	Synthetic CDN-mimetic STING agonist activating TBK1–IRF3 signaling; intratumoral administration	Increased IFN-β and chemokines; T-cell recruitment; modest single-agent activity, combination with anti-PD-1 explored.	Clinical	NCT03010176
SNX281	2020	Cancer	Small-molecule systemic STING agonist	Dose-dependent interferon signaling in preclinical/early clinical testing; development stopped by sponsor (not for safety).	Terminated	NCT04609579
TAK-676	2020	Cancer	Intravenous, systemically active STING agonist	Robust innate/adaptive immune activation; combination with pembrolizumab under evaluation.	Clinical	NCT04420884
GSK3745417	2019	Cancer	Small-molecule STING agonist (IV)	Induces IFN pathway activation; being tested alone and with anti-PD-1.	Clinical	NCT03843359
E7766	2019	Cancer	Macrocycle-bridged STING agonist (human-active)	Pharmacodynamic IFN signature and cytokine induction; early clinical disease control in IT study.	Clinical	NCT04144140
SYNB1891	2019	Cancer	Engineered *E. coli* Nissle producing CDNs to activate STING in APCs (intratumoral)	Local STING activation with innate immune priming; program terminated for business reasons.	Terminated	NCT04167137
BI-1703880	2022	Cancer	Next-generation systemic, non-CDN STING agonist (IV)	Early human data show pharmacodynamic activation; combination with anti-PD-1 in lead-in design.	Clinical	NCT05471856
SR-717	2019	Cancer	Non-nucleotide STING agonist; stabilizes active STING conformation	Increased IFN-β/chemokines; antitumor activity in mice; no registered clinical trial.	Preclinical	N/A

## 5 Conclusions

In summary, the cGAS–STING signaling pathway serves as a central hub in innate immune responses and plays a crucial role not only in anti-infective and antitumor immunity but also in the pathogenesis and progression of various orthopedic diseases. Evidence indicates that this pathway regulates the functions of osteoblasts, osteoclasts, and immune cells by sensing intracellular aberrant DNA and activating downstream type I interferons and inflammatory mediators, thereby influencing bone metabolism and tissue homeostasis. In OA, persistent activation of the pathway exacerbates cartilage inflammation and matrix degradation; In RA, persistent activation of the cGAS–STING pathway links aberrant self-DNA sensing with chronic synovial inflammation and joint destruction, highlighting its critical role in autoimmune bone erosion; in OP, dysregulated signaling contributes to the imbalance between bone formation and resorption; and in IVDD, the cGAS–STING pathway promotes cellular senescence, apoptosis, and inflammation, accelerating extracellular matrix degradation and annulus fibrosus damage. Notably, in bone tumors such as OS, the pathway exhibits a dual role. On one hand, activation of cGAS–STING enhances antitumor immunity by facilitating immune cell infiltration and tumor clearance; on the other hand, excessive or chronic activation may drive tumor-promoting inflammation and immune evasion. Collectively, these findings suggest that the cGAS–STING pathway functions as a “double-edged sword” in bone oncology, with its effects determined by tumor type, immune context, and microenvironmental cues. Future research should focus on delineating the spatiotemporal regulation of cGAS–STING signaling across different orthopedic conditions and bone tumors, elucidating its interplay with immune responses, bone metabolism, and disc homeostasis. Targeted modulation of this pathway—through small molecules, gene-editing tools, or biomaterial-based delivery systems—may open new avenues for precision therapy in orthopedic diseases and establish a theoretical and practical foundation for integrating the fields of immunology, bone metabolism, intervertebral disc biology, and oncology.
